# Platelet Detection Based on Improved YOLO_v3

**DOI:** 10.34133/2022/9780569

**Published:** 2022-09-14

**Authors:** Renting Liu, Chunhui Ren, Miaomiao Fu, Zhengkang Chu, Jiuchuan Guo

**Affiliations:** ^1^School of Information and Communication Engineering, University of Electronic Science and Technology, Chengdu 611731, China; ^2^School of Automation Engineering, University of Electronic Science and Technology, Chengdu 611731, China

## Abstract

Platelet detection and counting play a greatly significant role in medical field, especially in routine blood tests which can be used to judge blood status and diagnose related diseases. Therefore, platelet detection is valuable for diagnosing related blood diseases such as liver-related diseases. Blood analyzers and visual microscope counting were widely used for platelet detection, but the experimental procedure took nearly 20 minutes and can only be performed by a professional doctor. In recent years, technological breakthroughs in artificial intelligence have made it possible to detect red blood cells through deep learning methods. However, due to the inaccessibility of platelet datasets and the small size of platelets, deep learning-based platelet detection studies are almost nonexistent. In this paper, we carried out experiments for platelet detection based on commonly used object detection models, such as Single Shot Multibox Detector (SSD), RetinaNet, Faster_rcnn, and You Only Look Once_v3 (YOLO_v3). Compared with the other three models, YOLO_v3 can detect platelets more effectively. And we proposed three ideas for improvement based on YOLO_v3. Our study demonstrated that YOLO_v3 can be adopted for platelet detection accurately and in real time. We also implemented YOLO_v3 with multiscale fusion, YOLO_v3 with anchor box clustering, and YOLO_v3 with match parameter on our self-created dataset and, respectively, achieved 1.8% higher average precision (AP), 2.38% higher AP, and 2.05% higher AP than YOLO_v3. The comprehensive experiments revealed that YOLO_v3 with the improved ideas performs better in platelet detection than YOLO_v3.

## 1. Introduction

Routine blood examination [1] (commonly known as “complete blood count”) is one of the most important and commonly used examination items in hospitals at all levels. It is used for in-depth examination, preliminary data analysis, and exclusion of suspected diseases, which is of great significance for disease diagnosis. In blood routine, the platelet count is indispensable and can be achieved by platelet detection. Platelets are blood cells released into the blood by degranulation after megakaryocytes mature [2]. Their shapes are various and irregular. Normal platelets are 2-4 m in diameter. They are the smallest cells in the blood and have no nucleus. The main function of platelets is coagulation [3], that is, they would coagulate rapidly when a wound occurs, thereby reducing blood flow. If they decrease, there may be bleeding problems such as thrombocytopenic purpura, and when they increase, the blood is hypercoagulable and the potential for thrombosis is high [4]. In addition to the above cases, when the number of platelets is higher than the normal value, but the change is not very large, it is generally influenza or other mild illness, and it will return to the normal value as long as the condition improves. Significant reduction in platelets indicates that the body has coagulation problems, such as acute leukemia [5] and acute radiation sickness. At this time, timely treatment measures should be taken to prevent the further development of the disease.

The most commonly used cell detecting methods in medicine include the visual microscope detecting method [6], the blood cell analyzer method [7], and the detecting method based on machine learning [8]. Visual microscope detection method includes ordinary optical microscope [9] detection method and phase contrast microscope [10] method. But, optical microscopes have far less resolution than electron microscopes. In the ordinary optical microscope detection method, it is necessary to destroy red blood cells when processing the blood sample. However, red blood cells are not completely destroyed if diluted 20 or 40 times with red salt platelet thinner. The phase-contrast microscope counting method uses ammonium oxalate as the diluent [11], counts under a transparent microscope, and checks the count after taking pictures. The method is highly accurate, and platelets are easy to identify. Blood cell analyzers are typically implemented by principles based on impedance [12] or light scattering [13] measurements. However, these methods have shortcomings for platelet detection. On the one hand, these methods cannot distinguish platelet from erythrocyte debris and nonnegligible interfering particles. Because of their approximate size to platelet, these particles may be counted as platelets, resulting in less accurate platelet detection. On the other hand, large platelet is ignored in platelet detection algorithms used in hematology analyzers.

Although the above two methods are relatively accurate, they have some defects, such as the need for more skilled and professional experimental operators, because they greatly affect the accuracy of the experimental results. This experimental process takes a lot of time. When there are many experimental samples, a large number of professional inspectors are required. Finally, the subjectivity of the experimenter is an inevitable source of error in this experiment. With the rapid development of computer technology, the machine learning method came into being. More and more scholars have gradually applied the machine learning method to the field of medical testing and counting. Kaur et al. [14] proposed a combination method of Ostu and watershed to detect blood boards. The Secretion Curve Threshold [15] method can be used to separate red blood cells with the background. In general, the visual microscope detection method and blood cell analysis instrumentation are time-consuming and cannot be effectively used. The process of manually extracting characteristics based on machine-learning of blood cell detection methods is troublesome. For example, LBP (Local Binary Pattern) and HOG (histogram of oriented gradient) are artificially designed algorithms for feature extraction. Then, the feature constitutes the input vectors of classifier as feature vectors. This study solves the problems from the perspective of liberation and saving time.

Due to the rapid development of the image processing field in recent years, related technologies have also been introduced into medical image processing. This paper focuses on the detection of platelet by deep learning. The commonly used target detection methods are divided into single-stage and multistage [16] target detection. Given that a single-stage method is relatively simple to process than a multistage and better in real time, the core idea of multistage network is to convert detection into regression, which can complete target positioning and classification at one time. YOLO_v3 [17] draws on YOLO_v1 and YOLO_v2. Although there are not many innovations, it improves the detection accuracy while maintaining the speed advantage of the YOLO family, especially for small objects. The main idea of SSD [18] is to use Convolutional Neural Network (CNN) to extract features and evenly perform dense sampling at different positions of the picture. Different scales and aspect ratios can be used for sampling. RetinaNet [19] is a combined application of the original Feature Pyramid Network (FPN) and Fully Convolutional Network (FCN). YOLO_v4 [20] enhances the learning ability of CNN, remove computational bottlenecks, reduce the use of video memory, and speed up the inference speed of the network. YOLO_v4-tiny [21] is a simplified version of YOLO_v4, with less structure, but the speed is greatly increased.

In this paper, a single-stage target detection method is called YOLO_v3 [17]. Finally, YOLO_v3 outperforms multistage methods both in terms of accuracy and detection time of platelet detection.

The main contributions of this paper are as follows: establishing a platelet dataset, improving the network structure of YOLO_v3, improving the representation of matching parameters, improving the prior frameï¼Œand carrying out the experiments for platelet detection based on YOLO_v3, SSD (Single Shot Multibox Detector) [18], RetinaNet [19], and improved YOLO_v3.

## 2. Materials and Methods

To realize the platelet detection timely, the methods based on deep learning were applied to the detection task. In this section, we introduce our approach to implementing platelet detection, namely, the YOLO_v3 network which used the Darknet_53 as the feature extraction network. Then, we show the relative improvements based on YOLO_v3.

### 2.1. YOLO_v3

**Figure 1:**
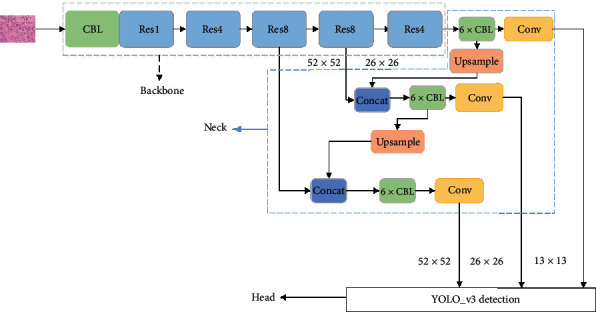
YOLO_v3's network.

YOLO_v3 is a single-stage CNN model for end-to-end detection. It consists of the backbone network Darknet_53 (except for the full connection layer) and a scale fusion network. The entire model structure is shown in Figure 1 [22].

In the figure above, “CBL” consists of three network layers: conv2d, Batch Normalization, and Leaky Relu. “ResX” denotes X residual blocks, and a residual block contains two convolution blocks and an “add” layer. Darknet_53 is mainly stacked with 5 subsample convolutions and residual blocks. The convolution layer subsamples images by varying the stride of the convolutional kernel to obtain feature maps with different sizes. The structure uses five residual blocks to complete the identity mapping and avoid gradient extinction as much as possible.

The “Neck” network is used for multiscale feature fusion [23] and outputs feature maps of different scales. If the size of the input image to the network is 416 × 416, the Neck's output would be feature maps of three sizes 13 × 13, 26 × 26, and 52 × 52. The feature map 13 × 13 is more suitable for large target detection, and 52 × 52 is more suitable for small target detection.

The “Head” architecture's output contains the class probability, the confidenceï¼Œand the boundary box coordinates.

### 2.2. Improved Ideas Based on YOLO_v3

#### 2.2.1. Improved Network Based on YOLO_v3

The shallow feature layer [24] of the neural network (close to the input layer) extracts low-level features. Low-level features are generalized and easy to express such as texture, color, and edges. High-level features are often complex, indescribable semantic information, such as blonde hair, ladybird wings, and colorful flowers. Therefore, it is not enough to directly use shallow or deep features, so high and low features need to be integrated into the head network.

For small-sized targets, the shallow features extracted by the network contain some of its details. However, as the number of layers deepens and the receptive field increases, the geometric details in the extracted features may disappear completely.

**Figure 2:**
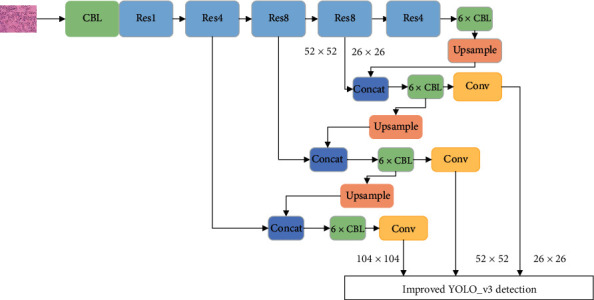
The improved YOLO_v3's network.

So we added a shallow feature layer 104 × 104 for the “Head” input in Figure 2 and abandoned the high-level feature 13 × 13; it is because the high-level feature 13 × 13 loses more original information. And the improved network is shown in the following figure:

#### 2.2.2. Anchor Boxes Using k-Means

YOLO_v3 adopted the k-means clustering mechanism to obtain anchors with the purpose of multiscale learning. The anchors are several boxes of different sizes obtained by statistics or clustering from the real boxes in the training set. After observation, it is found that the anchors generated by YOLO_v3 are closely connected with the data sets, and the size of the gap between platelets in this paper and the original data set (VOC2007) is too large. This paper improves it to produce anchor boxes of size [3, 6], [16, 18], [13, 19], [29, 29], [13, 25], [24, 30], [24, 39], [39, 42], and [45, 60] by k-means clustering method.

#### 2.2.3. Improved Match Parameter

In the experiments, there will be certain problems in the use of matching parameters between the real frame and the predicted frame, that is, if the two targets do not overlap, it will be 0, and the distance between the two targets will not be reflected at this time. In the case of the nonoverlapping target, if IOU was used as a matching parameter, the gradient would be 0 and cannot be optimized. IOU is defined in the following formula:

(1) $$\begin{array}{c} \text{IOU}\left(C,G\right)=\frac{\text{area}\left(C\right)\cap \text{area}\left(G\right)}{\text{area}\left(C\right)\cup \text{area}\left(G\right)}. \end{array}$$

IOU refers to the overlap ratio between the prior box *C* and the ground truth *G*. area(*C*) refers to the area of the prior box *C*, and area(*G*) refers to the area of the real box *G*. When the prior box is completely close to the real box, the value of IOU between the two boxes is 1.

Therefore, CIOU + IOU will be used as the matching parameter of the model, instead of the original matching parameter. CIOU + IOU means that the two values are added. Then, even though the real frame and the predicted frame do not overlap, the matching parameter still can measure how much the two boxes overlap. It is because that the CIOU takes into account the position information of the two boxes, such as the distance of central points of two boxes and the diagonal length of the smallest enclosing box covering two boxes. CIOU is shown in the following formula:

(2) $$\begin{array}{c} \text{CIOU}=\frac{\text{area}\left(b\right)\cap \text{area}\left(b^{gt}\right)}{\text{area}\left(b\right)\cup \text{area}\left(b^{gt}\right)}-\frac{\rho^{2}\left(b,b^{gt}\right)}{c^{2}}-\alpha \nu , \end{array}$$

where *b* represents the predicted frame, *b*^*gt*^ represents the real frame, area(*b*) represents the area of the predicted frame area, area(*b*^*gt*^) represents the area of the real frame area, *ρ*^2^(*b*, *b*^*gt*^) represents the center distance between the predicted frame and the real frame, *c* represents the diagonal distance of the smallest area that can include the predicted box and the ground-truth box, *α* is a parameter used for balance, and *ν* is a parameter used to measure the consistency of the aspect ratio.

### 2.3. Data Preparation

As platelet dataset is hard to get online, we exploited a homemade platelet dataset to carry out experiments to evaluate the proposed method.

Given the color of background proximity of platelets when whole blood cells are not processed, it is difficult to distinguish. Therefore, we colored the blood cells.

We identified the platelets under the microscope by observing the internal staining of the platelets when dynamically adjusting the focal length. Then, we used LabelImg to draw a rectangular box to surround the corresponding platelets to the most accurate degree; then, we can obtain the location information and category information of platelets for each picture. The total number of the dataset was 412; then, 296 were for training and 33 for validationï¼Œand 83 for testing.

#### 2.3.1. Blood Cell Image Acquisition System

**Figure 3:**
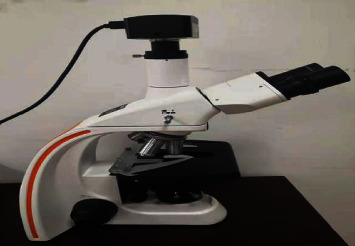
Microscope.

The image collection system in platelets was composed of two parts of hardware and software (shown as Table 1). The hardware is an electronic biooptical microscope, and the physical picture of the microscope is shown in Figure 3.

**Figure 4:**
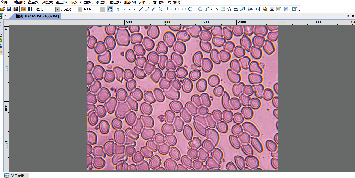
ImageView's visual interface.

Figure 3 shows a microscope with model L208-3M50. The microscope consists of the following parts: body tube, coarse adjustment, fine adjustment, objectives on nosepiece, limb, stage, joint, substage condenser, mirror, condenser adjustment, eyepiece, objective lens, and foot. We placed the blood smear on the stage and observed and saved the blood smear field map through the computer connected to the electronic eyepiece. We used ImageView as the software for the image collecting system, and the software interface is shown in Figure 4.

The center of Figure 4 is a view of a blood smear under a microscope, which is transmitted from the microscope's electronic eyepiece to the computer. The part of the content in blue font on the left is the relevant parameters for adjusting the screen, such as resolution, format, color mode, and color adjustment.

#### 2.3.2. Annotation of Blood Cell Image Dataset

**Figure 5:**
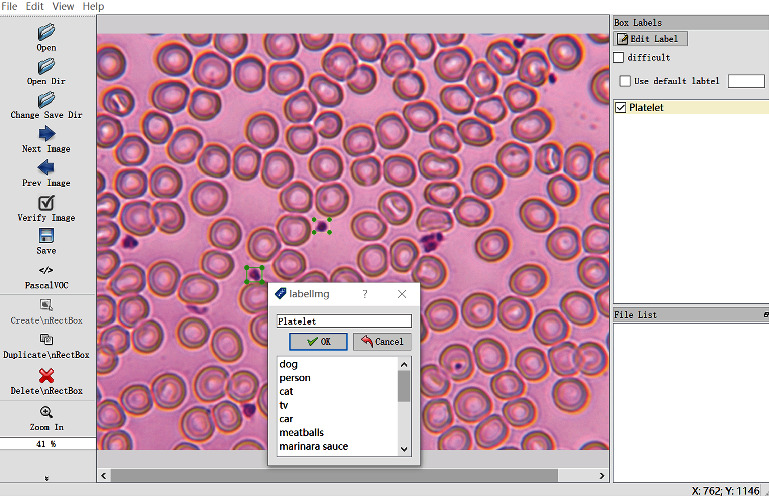
LabelImg interface.

The experiments for detecting platelet were carried out in a supervised learning manner; hence, the labeling information of the platelets was required. The labeling information was composed of the platelet boundary location information and the platelet category information and was obtained by utilizing labeling. The format of the final generated Extensible Markup Language file is the same as that of PASCAL VOC. The image annotation process is shown in Figure 5.

The left side of Figure 5 is the software operation options, such as “Open” which refers to opening a single image, “Open Dir” which refers to opening a folder, and “Change Save Dir” refers to the path where the image is saved, and “Create RectBox” draws a callout box. The right side of Figure 5 is an open image of blood cells. The green marked box in the figure is an annotation for platelets, and a “.xml” file will be generated later. The information in the file includes the location information, category, and name of the marked cells. Therefore, after completing the work of grasping the morphological characteristics of blood cells, collecting blood cells, and acquiring blood cell images, the preparation of the blood cell database is completed.

### 2.4. Experimental Environment and Parameter Setting

The experimental platform for the paper is equipped as follows:

Based on the above configuration, we implemented a total of five experiments employing Faster_rcnn, SDD, RetinaNet, YOLO_v3, and the proposed improved YOLO_v3 for the assessment of the performance of the platelet detection. The number of iterations of the proposed improved YOLO_v3 was set to 100. The learning rate (LR) was set to 0.001 when we first train the model and then was set to 0.0001 after 50 iterations. The LR was not fixed and adjusted at every iteration with the multiplication factor of 0.92. And the batch size was set to 4. The IOU threshold was set to 0.45 and the confidence threshold to 0.25 for obtaining a precise detection result.

### 2.5. Evaluation Metric

To quantitatively evaluate and compare five deep neural networks for detecting platelet, we utilized a variety of standard metrics frequently used to evaluate these methods including recall, precision, *F*1, and AP (average precision). And these metrics are obtained based on the confusion matrix in Table 2.

TP is true positive and indicates the number of positive samples classified as positive samples; FP is false positive and represents the number of negative samples that are misclassified as positive samples. FN is false negative and indicates the number of positive samples that are misclassified as negative samples. TN is true negative and indicates the number of negative samples that are classified as negative samples.

Precision is defined as follows:

(3) $$\begin{array}{c} \text{Precision}=\frac{\text{TP}}{\text{TP}+\text{FP}}. \end{array}$$

Recall is defined as follows:

(4) $$\begin{array}{c} \text{Recall}=\frac{\text{TP}}{\text{TP}+\text{FN}}. \end{array}$$

The *F*1 score can comprehensively evaluate the performance presented by the two indicators of precision and recall. When we create classifiers, we always make a compromise between recall and precision, and it is difficult to compare models with high recall and low precision compared to models with high precision but low recall. *F*1 score is a metric we can use to compare two models. *F*1 is defined as follows:

(5) $$\begin{array}{c} \frac{2}{F1}=\frac{1}{\text{precision}}+\frac{1}{\text{recall}},\\ F1=\frac{2\text{TP}}{2\text{TP}+\text{FP}+\text{FN}}. \end{array}$$

AP (average precision) is the precision across all elements of objects as defined in the following formula:

(6) $$\begin{array}{c} \text{AP}=\overset{M}{\underset{K=1}{\sum }}P\left(k\right)\Delta r\left(k\right), \end{array}$$

where *M* is the number of all targets predicted by the network, *P*(*k*) refers to the corresponding precision value when the model predicts *k* objects, and Δ*r*(*k*) represents the difference between the recall rates when the model predicts *k* − 1 to *k* object value.

## 3. Results

To ensure the fairness of experiments, the experiments were implemented under the same hardware environment and test images.

In order to well understand the effectiveness of YOLO_v3 used to detect platelet, we carried out experiments to compare the results of four methods. The experimental effects of different methods for platelet detection are shown in Table 3.

### 3.1. Comparative Experiment

To be specific, the average precision (AP) values, F1, precision, and recall of the four network models are shown in Table 3: YOLO_v3: 84.2%, SSD: 47.95%, RetinaNet: 20.37%, and Faster_rcnn: 33.75%, and the AP value of YOLO_v3 was 36.25%, 63.83%, and 50.45% higher than that of SSD, RetinaNet, and Faster_rcnn, respectively. The recall values of the four network models were as follows: YOLO_v3: 86.86%, SSD: 7.22%, RetinaNet: 15%, and Faster_rcnn: 74.17%, and the recall of YOLO_v3 was 79.64%, 71.86%, and 12.69% higher than that of SSD, RetinaNet, and Faster_rcnn, respectively. The precision values of the four network models were as follows: YOLO_v3: 85.31%, SSD: 85.51%, RetinaNet: 100%, and Faster_rcnn: 27.08%, and the recall of YOLO_v3 was -0.2%, -14.69%, and 58.23% higher than that of SSD, RetinaNet, and Faster_rcnn, respectively. The F1 values of the four network models were as follows: YOLO_v3: 86%, SSD: 13%, RetinaNet: 1%, and Faster_rcnn: 40%, and the F1 value of YOLO_v3 was 73%, 85%, and 46% higher than that of SSD, RetinaNet, and Faster_rcnn, respectively. The values of average precision (AP), precision, and F1 are demonstrated that the YOLO_v3 model outperforms the comparison networks. The total number of platelets SSD and RetinaNet recalled is much less than YOLO_v3. Thus, their higher recall compared to YOLO_v3 does not mean they are better than YOLO_v3. The detecting time of models were as follows: YOLO_v3: 2.85 s, SSD: 3.31 s, RetinaNet: 2.9 s, and Faster_rcnn: 2.95 s, which indicates that four networks can detect platelet in real time and YOLO_v3 achieved fastest detection of platelet compared other three methods. In general, YOLO_v3 is a competitive network for detecting platelet.

The above methods include single-stage target detection methods and a multistage target detection method (Faster_rcnn). As can be seen from Table 3, YOLO_v3 is more accurate in detecting platelet than the other three methods with a large margin and outperforms others in detecting speed.

The SSD network does not have a multiscale fusion module, and the features used in the prediction are separate deep features or shallow features. Therefore, it is not conducive to the detection of a small target platelet. Faster_rcnn was inferior to YOLO_v3 in platelet detection because the detection principle of Faster_rcnn requires candidate region screening, which can well separate the target from the background, while platelet is a small target, and there are not enough pixel features used for its learning. In addition, the pixel value of the platelet in the stained picture is close to the background, which makes Faster_rcnn unable to give full play to its advantages. RetinaNet detected platelet poorly because there are only three feature layers used to predict the platelet, and the shallow feature information contained is insufficient. YOLO_v4 and YOLO_v4-tiny can hardly learn the platelet's features because their improvements compared to YOLO_v3 are not suitable for platelet detection. The YOLO_v3 model has a multilayer feature map output for predicting platelet, and it also uses a multiscale fusion method, so that it can contain both shallow and deep feature information for the model to detect platelet.

**Figure 6:**
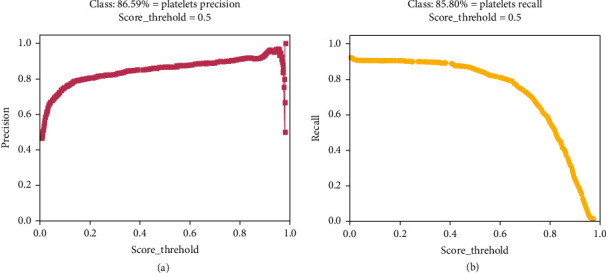
(a) Precision-threshold; (b) recall-threshold.

We carried out parametric experiments, and the experimental results are shown in Figure 6.

Figure 6(a) presents the relationship between threshold and precision. As can be seen from Figure 6(a), the accuracy rate will increase as the threshold (score_threshold) increases. When the threshold value is close to 1, the accuracy rate reaches the peak and begins to fall. Figure 6(b) shows the relationship between the recall rate and the threshold. It can be found that the recall rate will decrease as the threshold increases. It can be concluded that when the precision value is high, the conservative classifier will judge a positive sample as a positive sample when there is sufficient evidence but judge a positive sample as a negative sample when classifiers are not fully confident; then, recall rate at this time is low. Therefore, it is necessary to balance and integrate them. We would obtain balanceable results when the threshold is set to 0.5.

**Figure 7:**
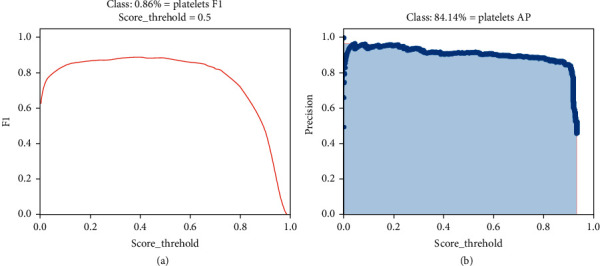
F1-threshold and P-R diagrams. (a) F1-threshold; (b) P-R diagram.

Figure 7(a) shows the relationship between F1 and the threshold. It can be seen that F1 increases from the beginning and starts to decrease when the threshold is 0.5, that is, when the threshold is 0.5, the model would obtain the most ideal effect when detecting platelet. Figure 7(b) presents the precision (*P*, precision) graph under the recall rate (recall). It can be seen from the graph that precision and recall are a pair of contradictory values. Thus, there is no possibility that both are ideal.

### 3.2. Ablation Study

To validate the practicality of improved_YOLO_v3 for detecting platelet, ablation experiments were implemented and experiment results are shown in Table 4. The results of a row are generated when the header corresponding to ✓ occurs but the header corresponding to × does not.

#### 3.2.1. Impact of Multiscale Fusion

First of all, we studied the impact of multiscale fusion on our model. To avoid ambiguity, we treated the header as the first row in the table. The second row in Table 4 shows the experimental results of YOLO_v3, and the third row shows the results of the model with new multiscale fusion architecture. The third row's AP, precision, and F1 are both larger than the second row's. It can be indicated that the new multiscale fusion architecture was helpful to improve the average precision for detecting platelet. The YOLO_v3 with the multiscale fusion architecture achieves better effects of detection because the multiscale fusion architecture strengthened the extraction of information from the shallow feature layer; the information from shallow layers is more suitable for detecting small targets (platelet belong to small targets).

#### 3.2.2. Impact of Anchor Box Clustering

We also analysed the effect of anchor box clustering on our proposed method. The fourth row in Table 4 presents the results of YOLO_v3 with anchor box clustering. The fourth row's AP, precision, and F1 are both larger than the second row's. The fourth row's recall is close to the second row's. Thus, we can conclude that anchor box clustering contributed to enhancing the performance for detecting platelet. Since anchor box clustering is approximate in size to the platelet dataset in this paper, the model with anchor box clustering can locate the position of platelet with less error.

#### 3.2.3. Impact of Match Parameter

We conducted experiments to discuss the contribution of the match parameter to the proposed method. The relative results are shown in the last three rows of Table 4. The results of the fifth row show that match parameter is of benefit to improving the precision of detecting platelet. In addition, we carried out experiments of YOLO_v3 with match parameter and anchor box clustering. The sixth row presents the largest values of AP, precision, recall, and F1 and approximative value of time compared with other rows. Consequently, we believed that this method can achieve better performance for platelet detection. The last row shows that YOLO_v3 with multiscale fusion architecture and matching parameter achieved platelet detection with lower average accuracy than YOLO_v3 because the matching parameter component contradicts the multiscale fusion architecture.

**Figure 8:**
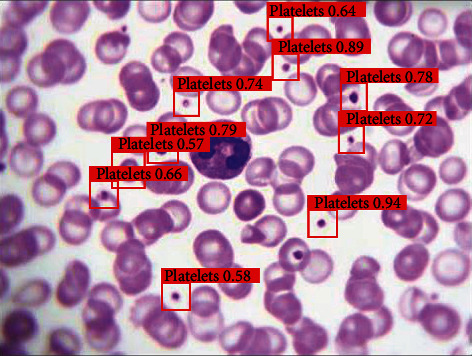
Prediction results.

Thus, YOLO_v3 with anchor box clustering and match parameter is our final chosen model.  Figure 8 shows how the final chosen model works when actually performing the recognition task.

Figure 8 shows our final chosen model's detection results for a blood cell image. The image downloaded randomly is a newly taken image that is not in the original dataset. Our model judges that there is a platelet in each red rectangle. The numbers in red represent our model's confidence in making a judgment. We can find that YOLO_v3 with anchor box clustering and match parameters almost has detected all platelets in the image taken randomly from the web, which proved the robustness and accuracy of our method.

## 4. Discussion

In this paper, we realized the platelet detection task on the whole via the YOLO_v3 network used a small number of platelet images that are manually annotated. Our comparative experiments show that YOLO_v3 can achieve better detection results that close to clinical requirements. And we proposed three ideas to enhance the detection performance. The experimental results demonstrated that each improvement idea improves the detection effect compared with YOLO_v3. Among them, simultaneous changes in match parameter and anchor box clustering can obtain the best AP (87.3%) in platelet detection and the AP lifts by 3.11%. Moreover, we achieved real-time platelet detection at the same time and can significantly lighten the load of doctors. Comprehensive experiments on our self-created platelet dataset indicated the validity and superiority of our method.

## Figures and Tables

**Table 1:** Experimental platform.

CPU	Ryzen 5 3600X
Memory	16 GB
GPU	NVIDIA GeForce RTX 2060
Programming language	Python3.6
Main library	Pytorch1.6

**Table 2:** Confusion matrix.

True class	Positive	Negative
Predicted class		
Negative	FP	TN
Positive	TP	FN

**Table 3:** Comparative experiments of platelet detection based on different methods.

Method	AP	*F*1	Precision	Recall	Time
RetinaNet	20.37%	1%	100%	15%	2.9 s
SSD	47.95%	13%	85.51%	7.22%	3.31 s
Faster_rcnn	33.75%	40%	27.08%	74.17%	2.95 s
YOLO_v3	84.20%	86%	85.31%	86.86%	2.85 s
YOLO_v4	13.65%	14%	41.43%	8.76%	3.0 s
YOLO_v4-tiny	24.67%	38%	48.27%	31.57%	2.95 s

**Table 4:** Experimental results of the improved method based on YOLO_v3.

BaseNet	Multiscale fusion	Anchor box clustering	Match parameter	AP	F1	Precision	Recall	Time
YOLO_v3	×	×	×	84.20%	86%	85.31%	86.86%	2.85 s
YOLO_v3	✓	×	×	86%	87%	88.51%	86.10%	3.654 s
YOLO_v3	×	✓	×	86.58%	87%	87.11%	86.25%	2.688 s
YOLO_v3	×	×	✓	86.25%	87%	86.73%	86.86%	2.721 s
YOLO_v3	×	✓	✓	87.31%	88%	89.06%	87.31%	4.07 s
YOLO_v3	✓	×	✓	80.63%	84%	84.4%	83.12%	3.32 s

## Data Availability

The data used to support the findings of this study are available from the corresponding author upon request.
